# Supplementary material for: Desmin Knock-Out Cardiomyopathy: A Heart on the Verge of Metabolic Crisis
Source: Int J Mol Sci. 2022 Oct 10;23(19):12020. doi: 10.3390/ijms231912020 (PMC9570457; doi:10.3390/ijms231912020)
Supplement: Supplementary file 1 [file ijms-23-12020-s001.zip › 2022-09-28_DesKO-Mito_Figure-S1_v2.pdf]

Supplementary Figure S1.

Bubble plot illustration of the “Gene Ontology (GO) term Cellular compartment (CC)” and “Kyoto Encyclopedia of Genes and Genomes (KEGG) pathway” enrichments derived from hierarchical clustering of all quantitated proteins; see also Figure 6A and Table S1.

Terms and pathways were sorted alphabetically, dot sizes indicate fold enrichment values, color gradient corresponds to corrected p-values (Benjamini-Hochberg), and highest fold enrichment scores were sorted by rank (Rank Fold Enrichment).

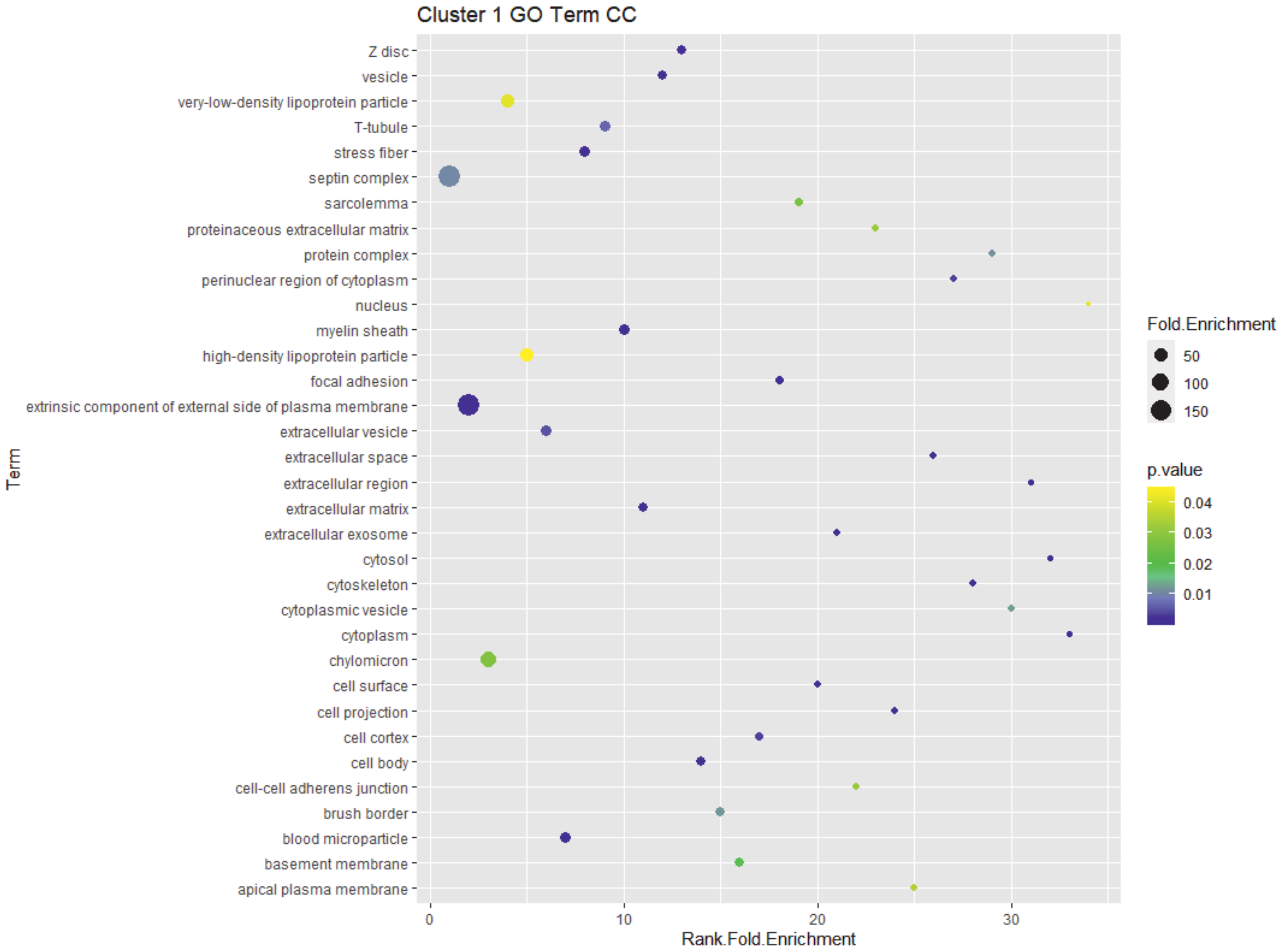
Cluster 1 GO Term CC
Z disc
vesicle
very-low-density lipoprotein particle
T-tubule
stress fiber
septin complex
sarcolemma
proteinaceous extracellular matrix
protein complex
perinuclear region of cytoplasm
nucleus
myelin sheath
high-density lipoprotein particle
focal adhesion
extrinsic component of external side of plasma membrane
extracellular vesicle
extracellular space
extracellular region
extracellular matrix
extracellular exosome
cytosol
cytoskeleton
cytoplasmic vesicle
cytoplasm
chylomicron
cell surface
cell projection
cell cortex
cell body
cell-cell adherens junction
brush border
blood microparticle
basement membrane
apical plasma membrane
Term
0
10
20
30
Rank.Fold.Enrichment
Fold.Enrichment
50
100
150
p.value
0.04
0.03
0.02
0.01
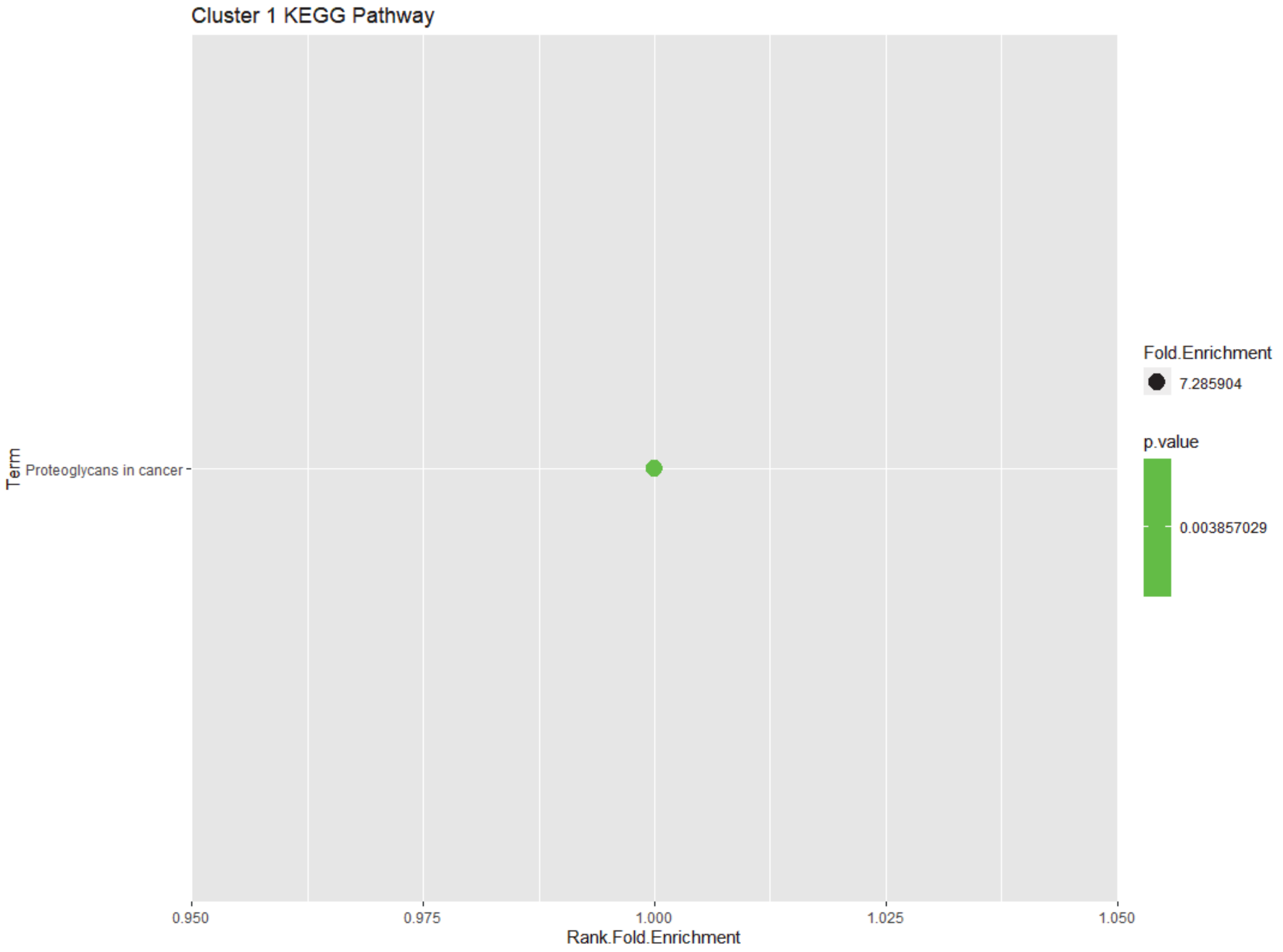
Cluster 1 KEGG Pathway
Term
Proteoglycans in cancer
0.950
0.975
1.000
1.025
1.050
Rank.Fold.Enrichment
Fold.Enrichment
7.285904
p.value
0.003857029

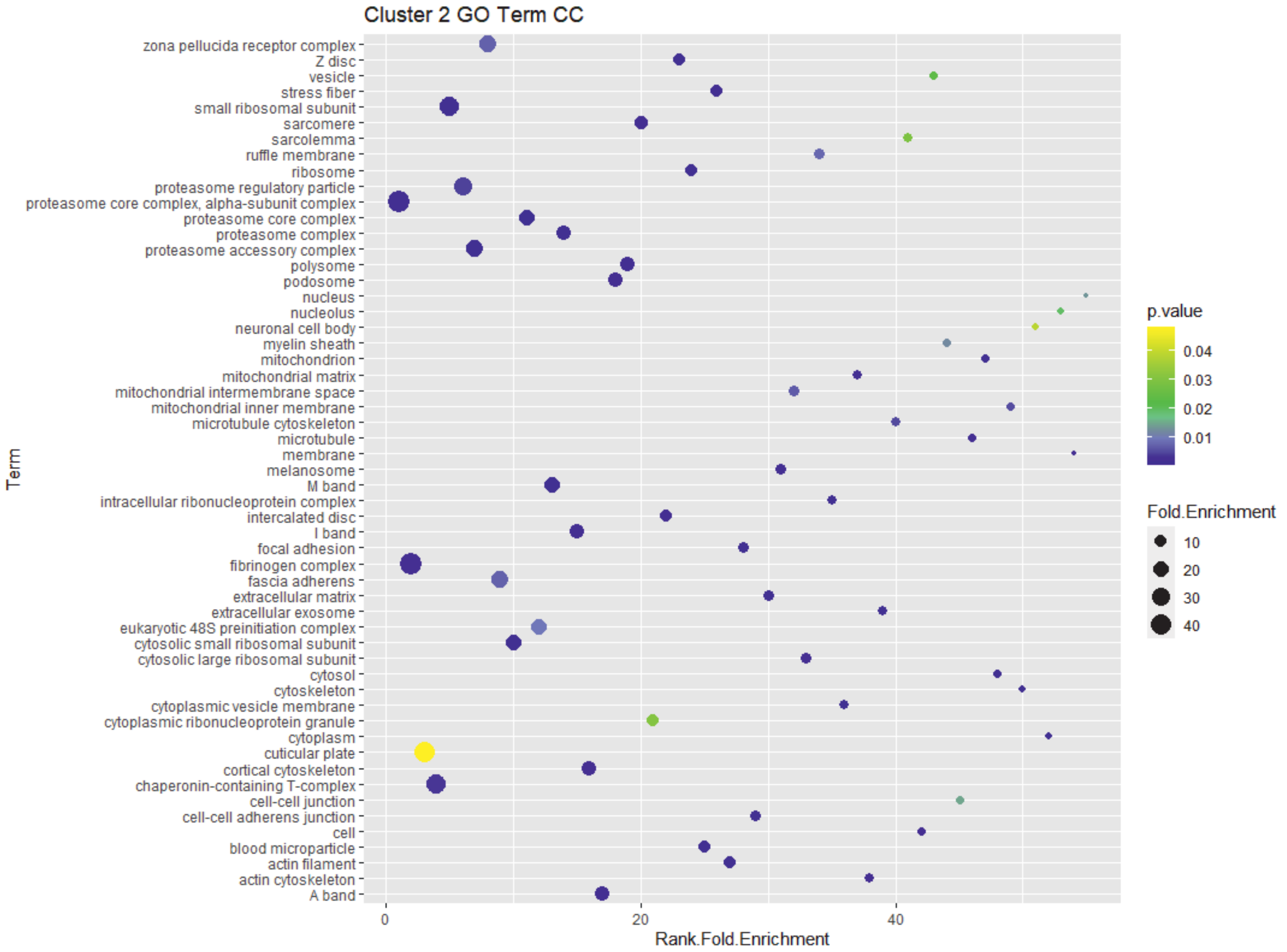

Cluster 2 GO Term CC
zona pellucida receptor complex
Z disc
vesicle
stress fiber
small ribosomal subunit
sarcomere
sarcolemma
ruffle membrane
ribosome
proteasome regulatory particle
proteasome core complex, alpha-subunit complex
proteasome core complex
proteasome complex
proteasome accessory complex
polysome
podosome
nucleus
nucleolus
neuronal cell body
myelin sheath
mitochondrion
mitochondrial matrix
mitochondrial intermembrane space
mitochondrial inner membrane
microtubule cytoskeleton
microtubule
membrane
melanosome
M band
intracellular ribonucleoprotein complex
intercalated disc
I band
focal adhesion
fibrinogen complex
fascia adherens
extracellular matrix
extracellular exosome
eukaryotic 48S preinitiation complex
cytosolic small ribosomal subunit
cytosolic large ribosomal subunit
cytosol
cytoskeleton
cytoplasmic vesicle membrane
cytoplasmic ribonucleoprotein granule
cytoplasm
cuticular plate
cortical cytoskeleton
chaperonin-containing T-complex
cell-cell junction
cell-cell adherens junction
cell
blood microparticle
actin filament
actin cytoskeleton
A band
Term
0
20
40
Rank.Fold.Enrichment
p.value
0.04
0.03
0.02
0.01
Fold.Enrichment
10
20
30
40

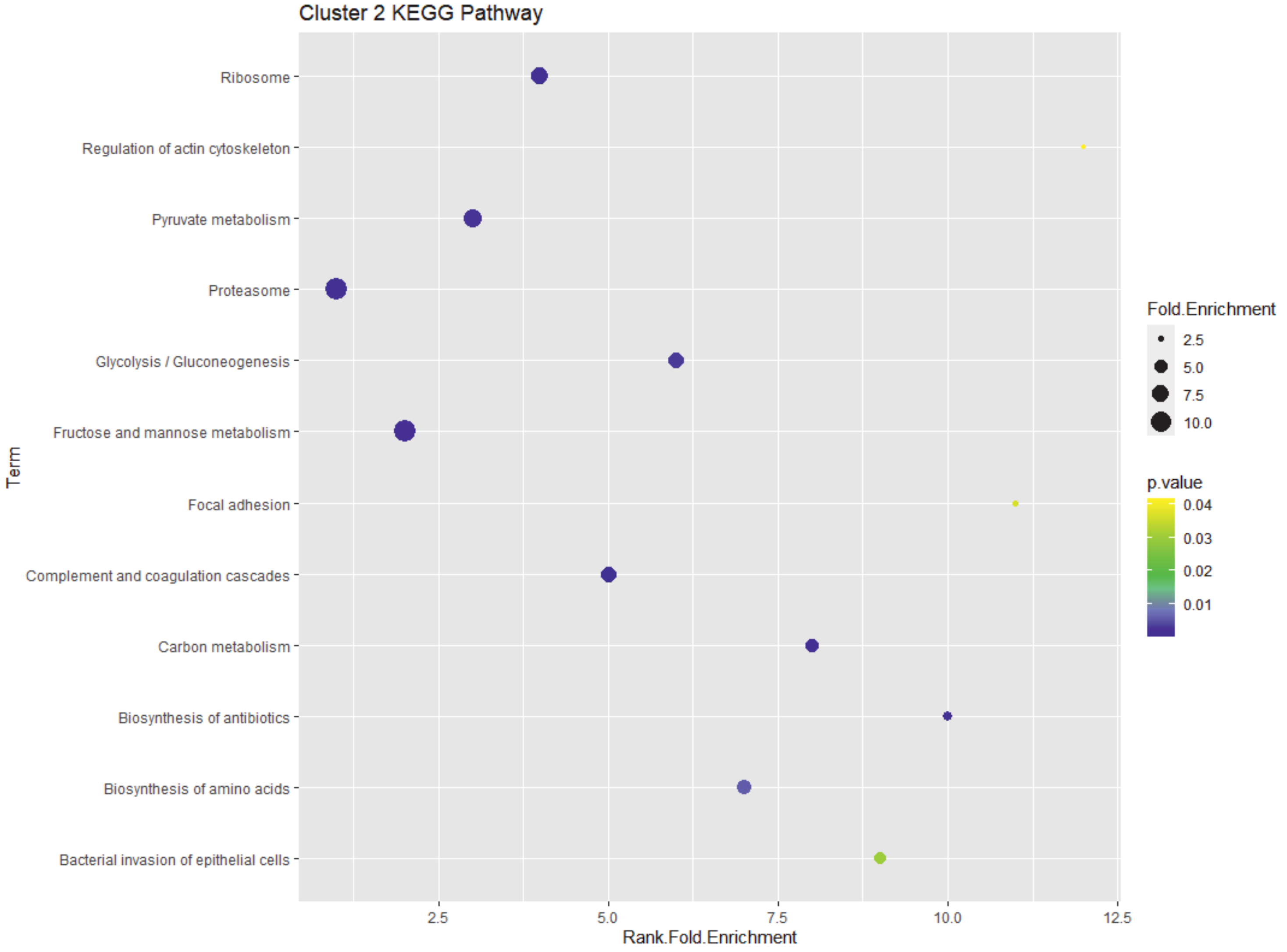

Cluster 2 KEGG Pathway
Ribosome
Regulation of actin cytoskeleton
Pyruvate metabolism
Proteasome
Glycolysis / Gluconeogenesis
Fructose and mannose metabolism
Focal adhesion
Complement and coagulation cascades
Carbon metabolism
Biosynthesis of antibiotics
Biosynthesis of amino acids
Bacterial invasion of epithelial cells
Term
2.5
5.0
7.5
10.0
12.5
Rank.Fold.Enrichment
Fold.Enrichment
2.5
5.0
7.5
10.0
p.value
0.04
0.03
0.02
0.01

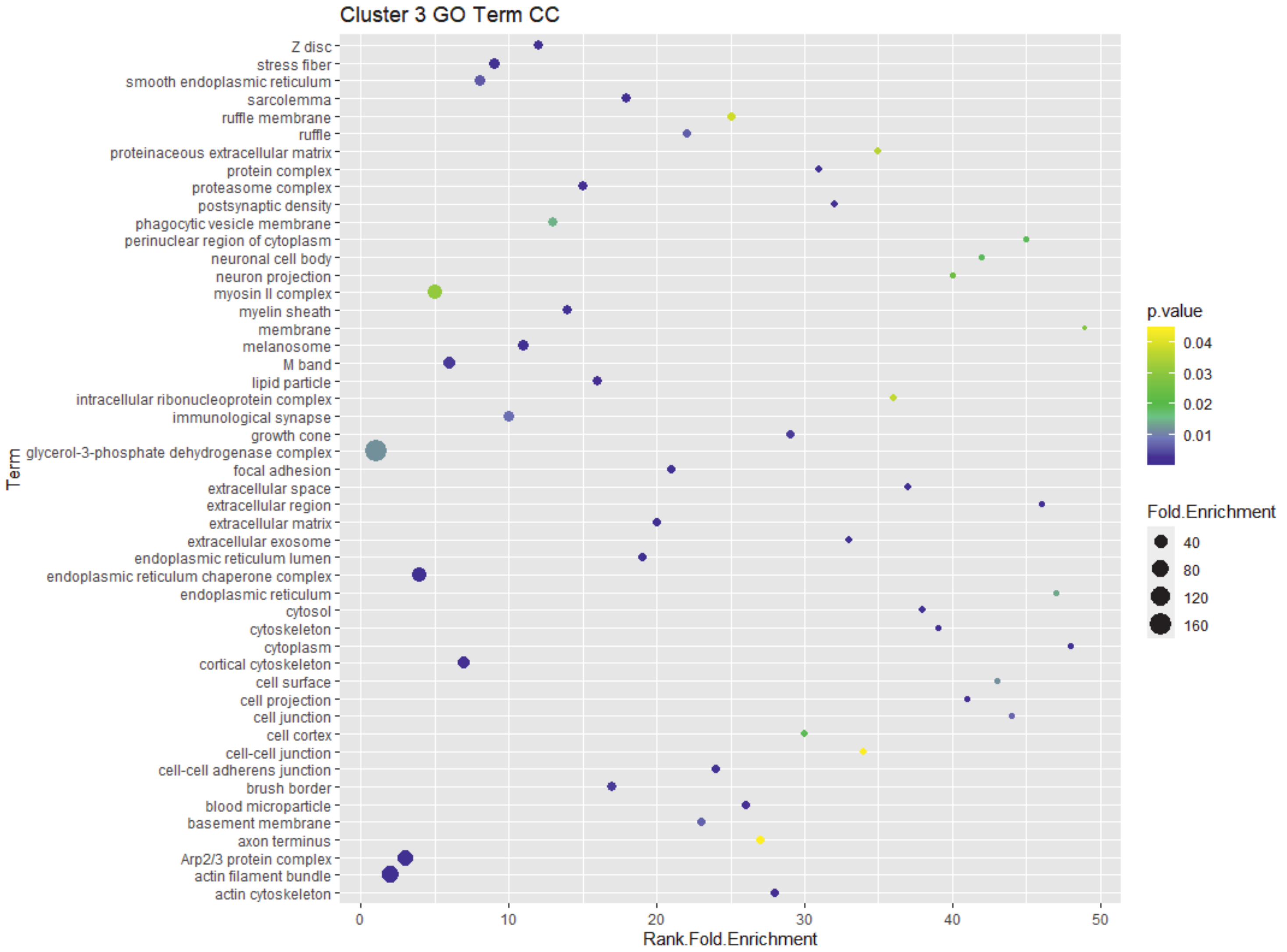
Cluster 3 GO Term CC
Z disc
stress fiber
smooth endoplasmic reticulum
sarcolemma
ruffle membrane
ruffle
proteinaceous extracellular matrix
protein complex
proteasome complex
postsynaptic density
phagocytic vesicle membrane
perinuclear region of cytoplasm
neuronal cell body
neuron projection
myosin II complex
myelin sheath
membrane
melanosome
M band
lipid particle
intracellular ribonucleoprotein complex
immunological synapse
growth cone
glycerol-3-phosphate dehydrogenase complex
focal adhesion
extracellular space
extracellular region
extracellular matrix
extracellular exosome
endoplasmic reticulum lumen
endoplasmic reticulum chaperone complex
endoplasmic reticulum
cytosol
cytoskeleton
cytoplasm
cortical cytoskeleton
cell surface
cell projection
cell junction
cell cortex
cell-cell junction
cell-cell adherens junction
brush border
blood microparticle
basement membrane
axon terminus
Arp2/3 protein complex
actin filament bundle
actin cytoskeleton
Term
0
10
20
30
40
50
Rank.Fold.Enrichment
p.value
0.04
0.03
0.02
0.01
Fold.Enrichment
40
80
120
160

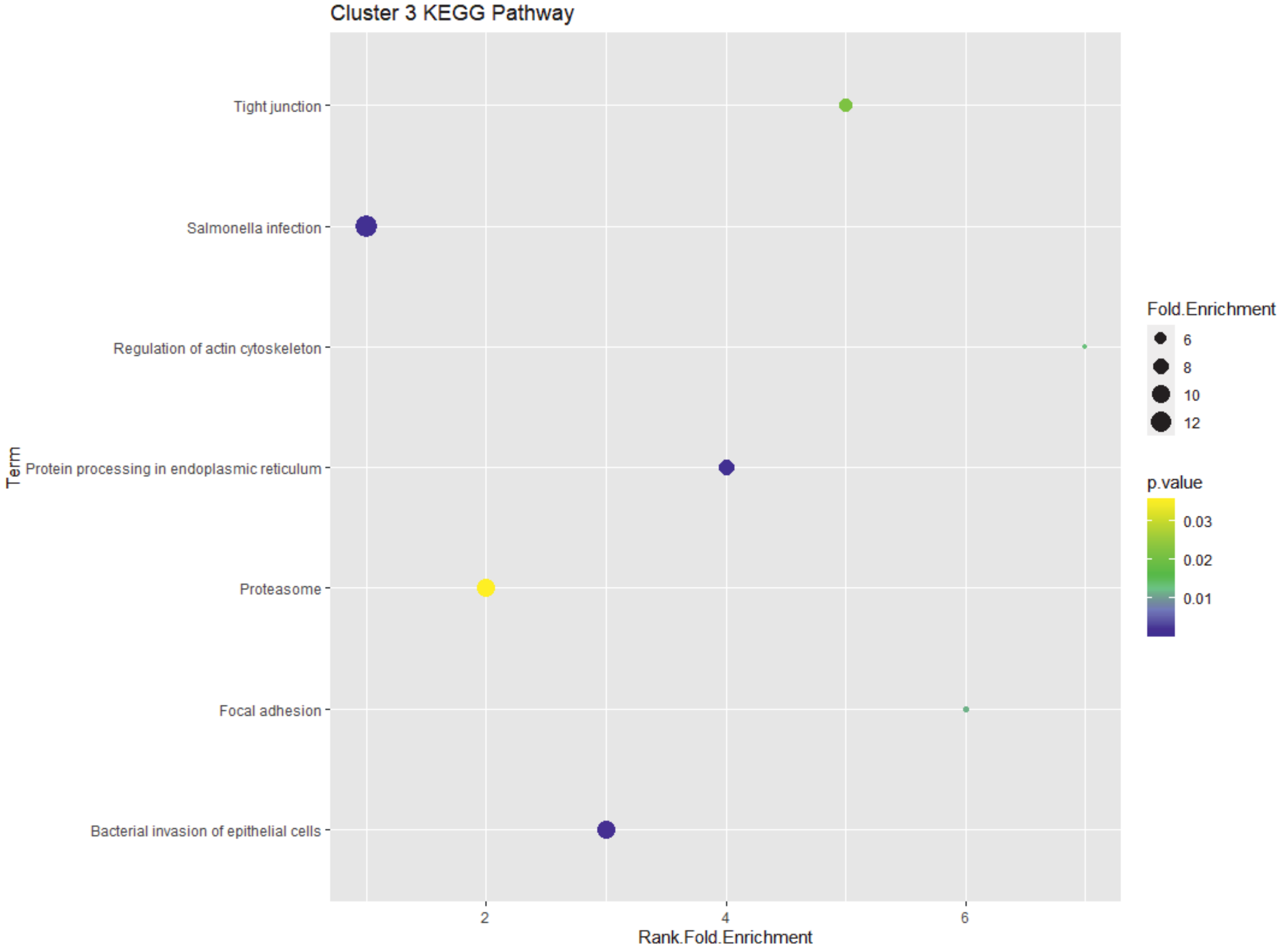
Cluster 3 KEGG Pathway
Tight junction
Salmonella infection
Regulation of actin cytoskeleton
Protein processing in endoplasmic reticulum
Proteasome
Focal adhesion
Bacterial invasion of epithelial cells
Term
2
4
6
Rank.Fold.Enrichment
Fold.Enrichment
6
8
10
12
p.value
0.03
0.02
0.01

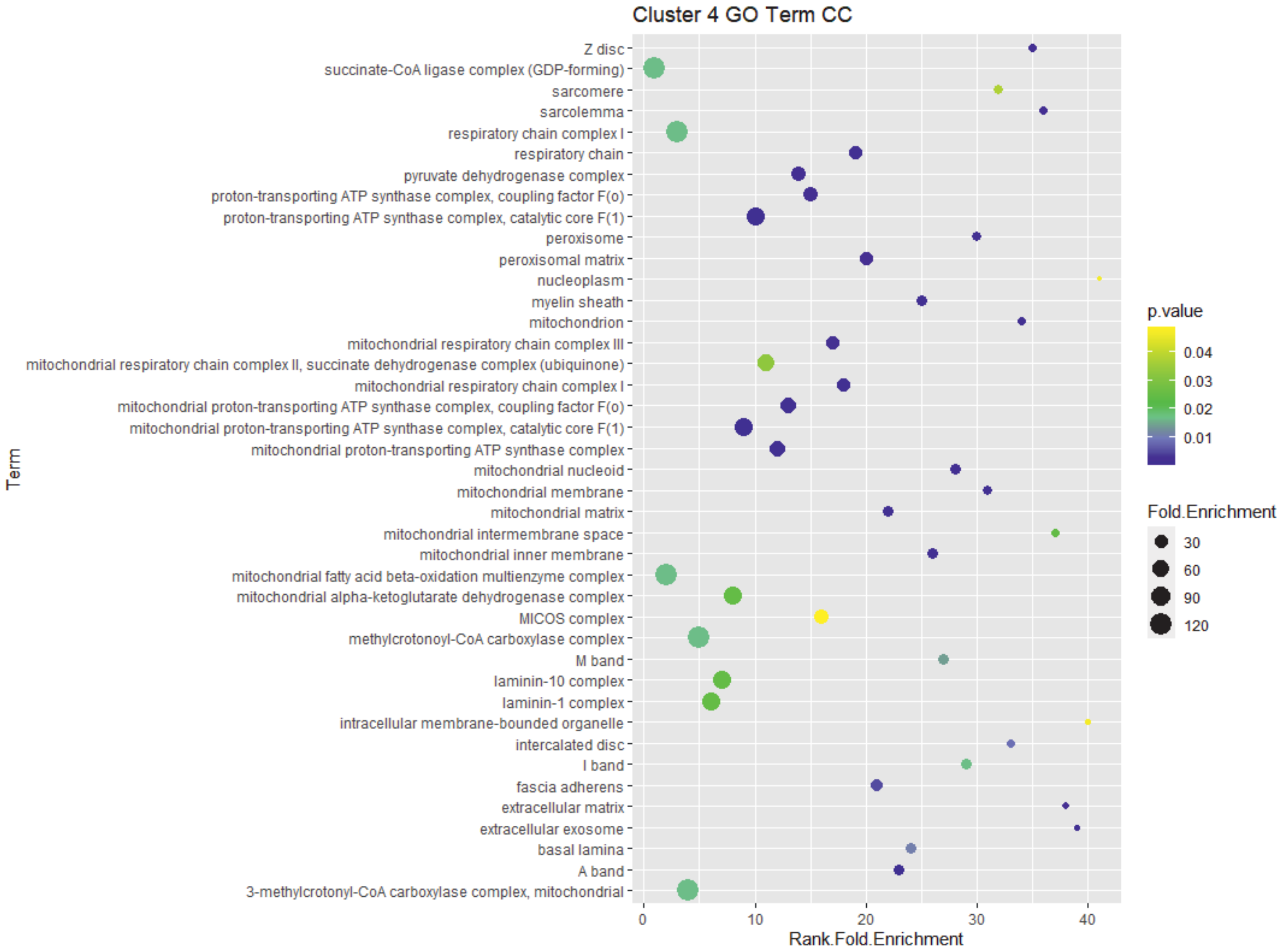

Cluster 4 GO Term CC
Z disc
succinate-CoA ligase complex (GDP-forming)
sarcomere
sarcolemma
respiratory chain complex I
respiratory chain
pyruvate dehydrogenase complex
proton-transporting ATP synthase complex, coupling factor F(o)
proton-transporting ATP synthase complex, catalytic core F(1)
peroxisome
peroxisomal matrix
nucleoplasm
myelin sheath
mitochondrion
mitochondrial respiratory chain complex III
mitochondrial respiratory chain complex II, succinate dehydrogenase complex (ubiquinone)
mitochondrial respiratory chain complex I
mitochondrial proton-transporting ATP synthase complex, coupling factor F(o)
mitochondrial proton-transporting ATP synthase complex, catalytic core F(1)
mitochondrial proton-transporting ATP synthase complex
mitochondrial nucleoid
mitochondrial membrane
mitochondrial matrix
mitochondrial intermembrane space
mitochondrial inner membrane
mitochondrial fatty acid beta-oxidation multienzyme complex
mitochondrial alpha-ketoglutarate dehydrogenase complex
MICOS complex
methylcrotonoyl-CoA carboxylase complex
M band
laminin-10 complex
laminin-1 complex
intracellular membrane-bounded organelle
intercalated disc
I band
fascia adherens
extracellular matrix
extracellular exosome
basal lamina
A band
3-methylcrotonyl-CoA carboxylase complex, mitochondrial
Term
0
10
20
30
40
Rank.Fold.Enrichment
p.value
0.04
0.03
0.02
0.01
Fold.Enrichment
30
60
90
120

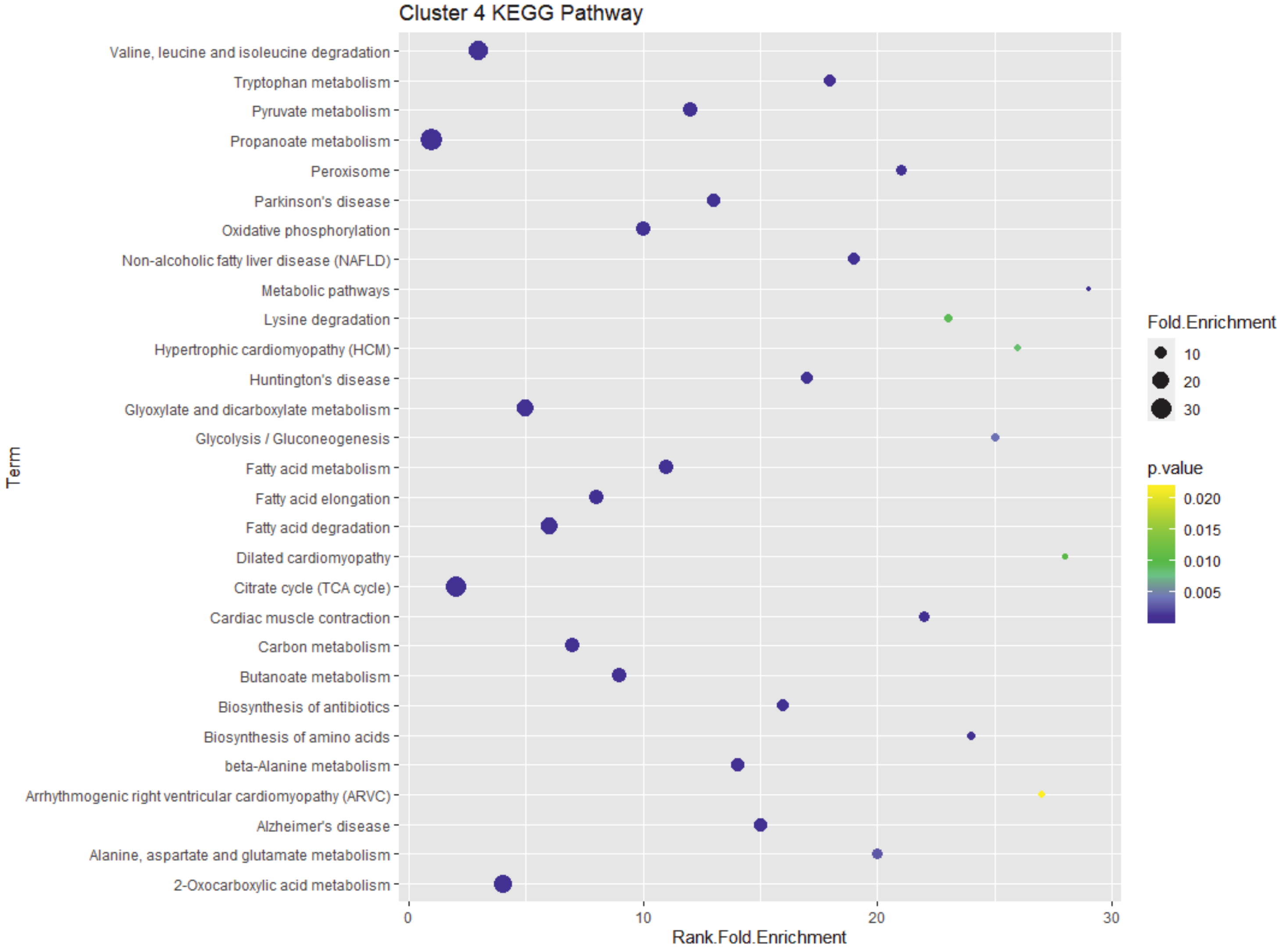

Cluster 4 KEGG Pathway
Valine, leucine and isoleucine degradation
Tryptophan metabolism
Pyruvate metabolism
Propanoate metabolism
Peroxisome
Parkinson's disease
Oxidative phosphorylation
Non-alcoholic fatty liver disease (NAFLD)
Metabolic pathways
Lysine degradation
Hypertrophic cardiomyopathy (HCM)
Huntington's disease
Glyoxylate and dicarboxylate metabolism
Glycolysis / Gluconeogenesis
Fatty acid metabolism
Fatty acid elongation
Fatty acid degradation
Dilated cardiomyopathy
Citrate cycle (TCA cycle)
Cardiac muscle contraction
Carbon metabolism
Butanoate metabolism
Biosynthesis of antibiotics
Biosynthesis of amino acids
beta-Alanine metabolism
Arrhythmogenic right ventricular cardiomyopathy (ARVC)
Alzheimer's disease
Alanine, aspartate and glutamate metabolism
2-Oxocarboxylic acid metabolism
Term
0
10
20
30
Rank.Fold.Enrichment
Fold.Enrichment
10
20
30
p.value
0.020
0.015
0.010
0.005

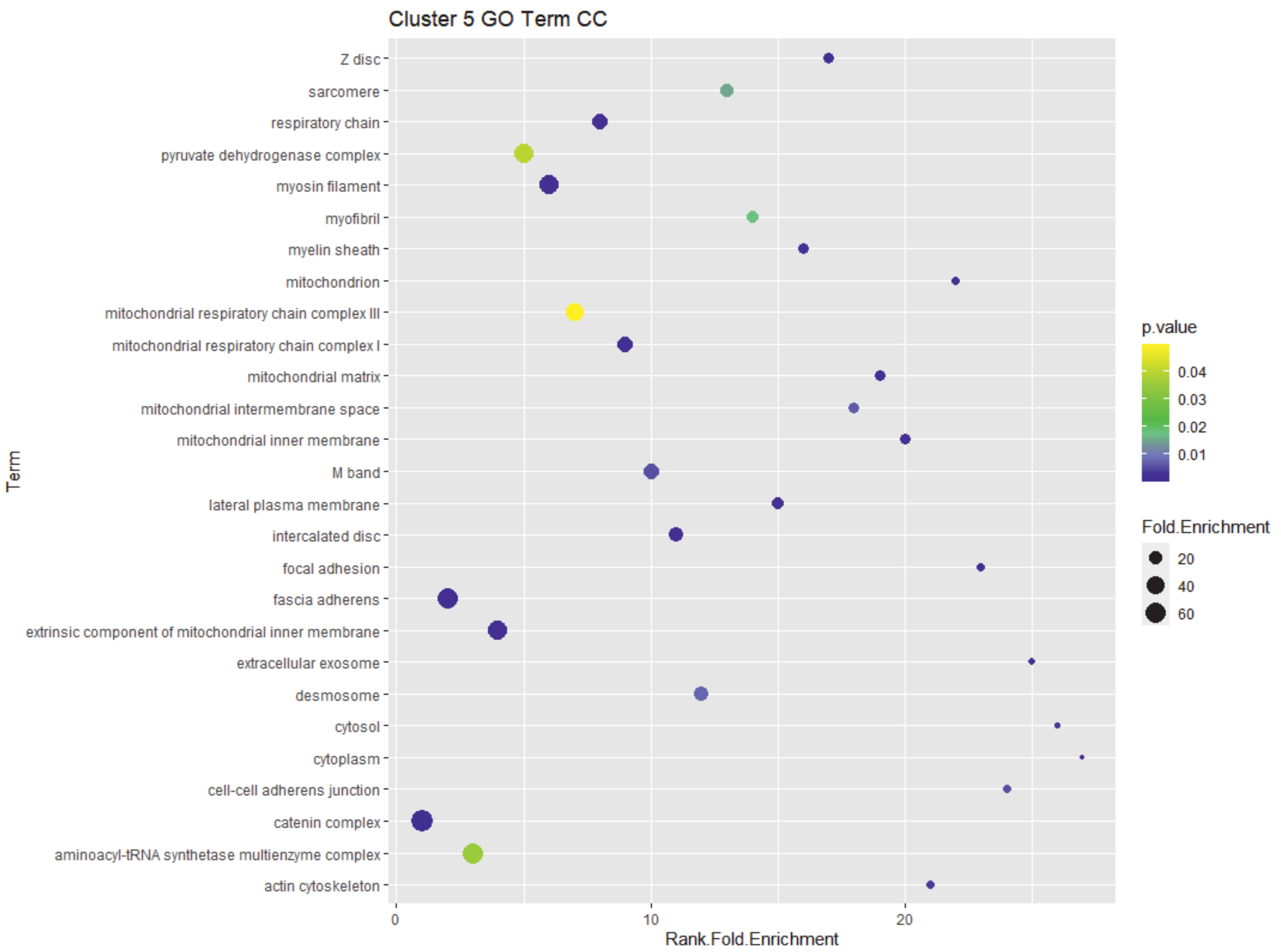
Cluster 5 GO Term CC
Z disc
sarcomere
respiratory chain
pyruvate dehydrogenase complex
myosin filament
myofibril
myelin sheath
mitochondrion
mitochondrial respiratory chain complex III
mitochondrial respiratory chain complex I
mitochondrial matrix
mitochondrial intermembrane space
mitochondrial inner membrane
M band
lateral plasma membrane
intercalated disc
focal adhesion
fascia adherens
extrinsic component of mitochondrial inner membrane
extracellular exosome
desmosome
cytosol
cytoplasm
cell-cell adherens junction
catenin complex
aminoacyl-tRNA synthetase multienzyme complex
actin cytoskeleton
Term
0
10
20
Rank.Fold.Enrichment
p.value
0.04
0.03
0.02
0.01
Fold.Enrichment
20
40
60

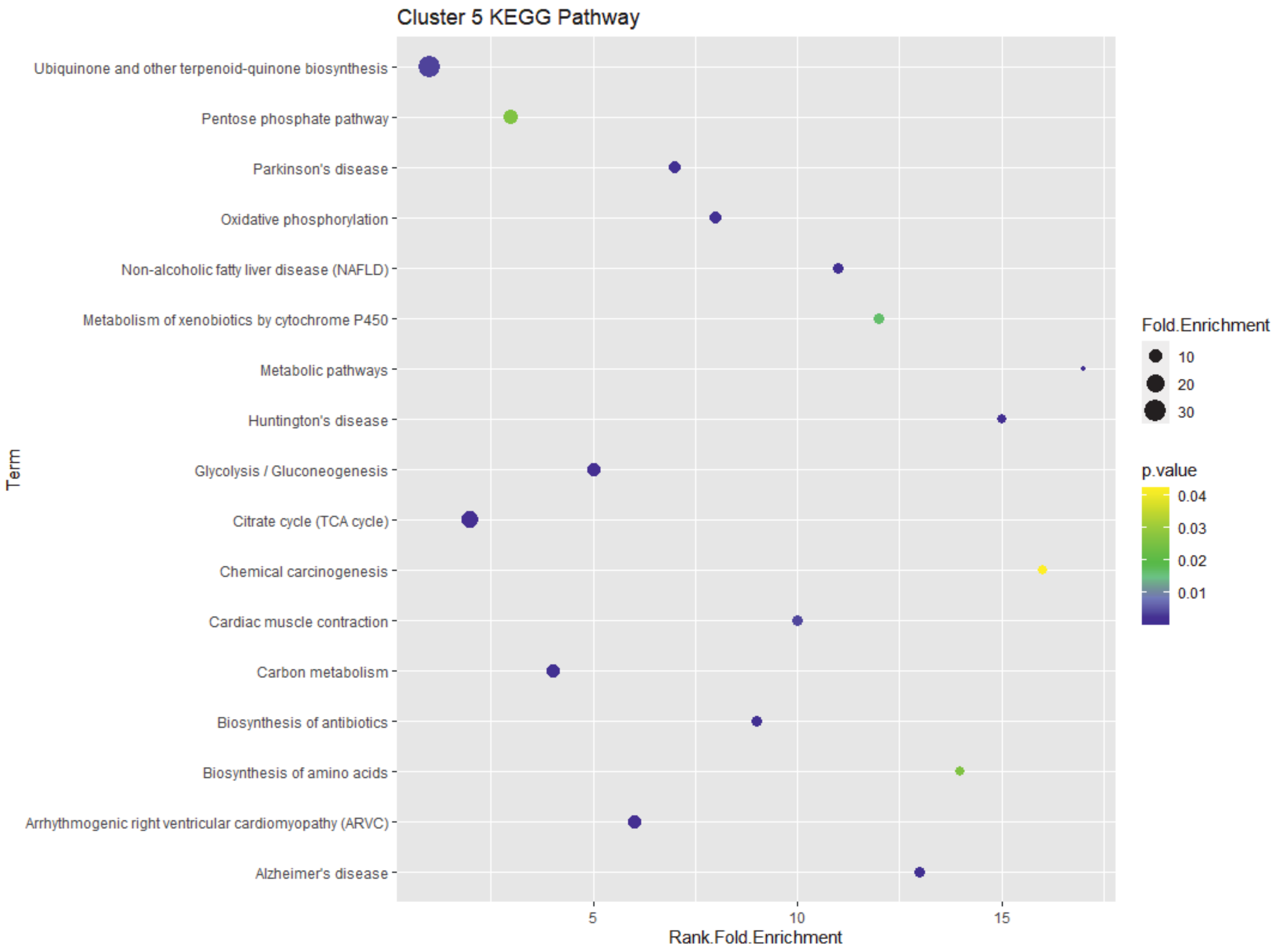
Cluster 5 KEGG Pathway
Ubiquinone and other terpenoid-quinone biosynthesis
Pentose phosphate pathway
Parkinson's disease
Oxidative phosphorylation
Non-alcoholic fatty liver disease (NAFLD)
Metabolism of xenobiotics by cytochrome P450
Metabolic pathways
Huntington's disease
Glycolysis / Gluconeogenesis
Citrate cycle (TCA cycle)
Chemical carcinogenesis
Cardiac muscle contraction
Carbon metabolism
Biosynthesis of antibiotics
Biosynthesis of amino acids
Arrhythmogenic right ventricular cardiomyopathy (ARVC)
Alzheimer's disease
Term
5
10
15
Rank.Fold.Enrichment
Fold.Enrichment
10
20
30
p.value
0.04
0.03
0.02
0.01

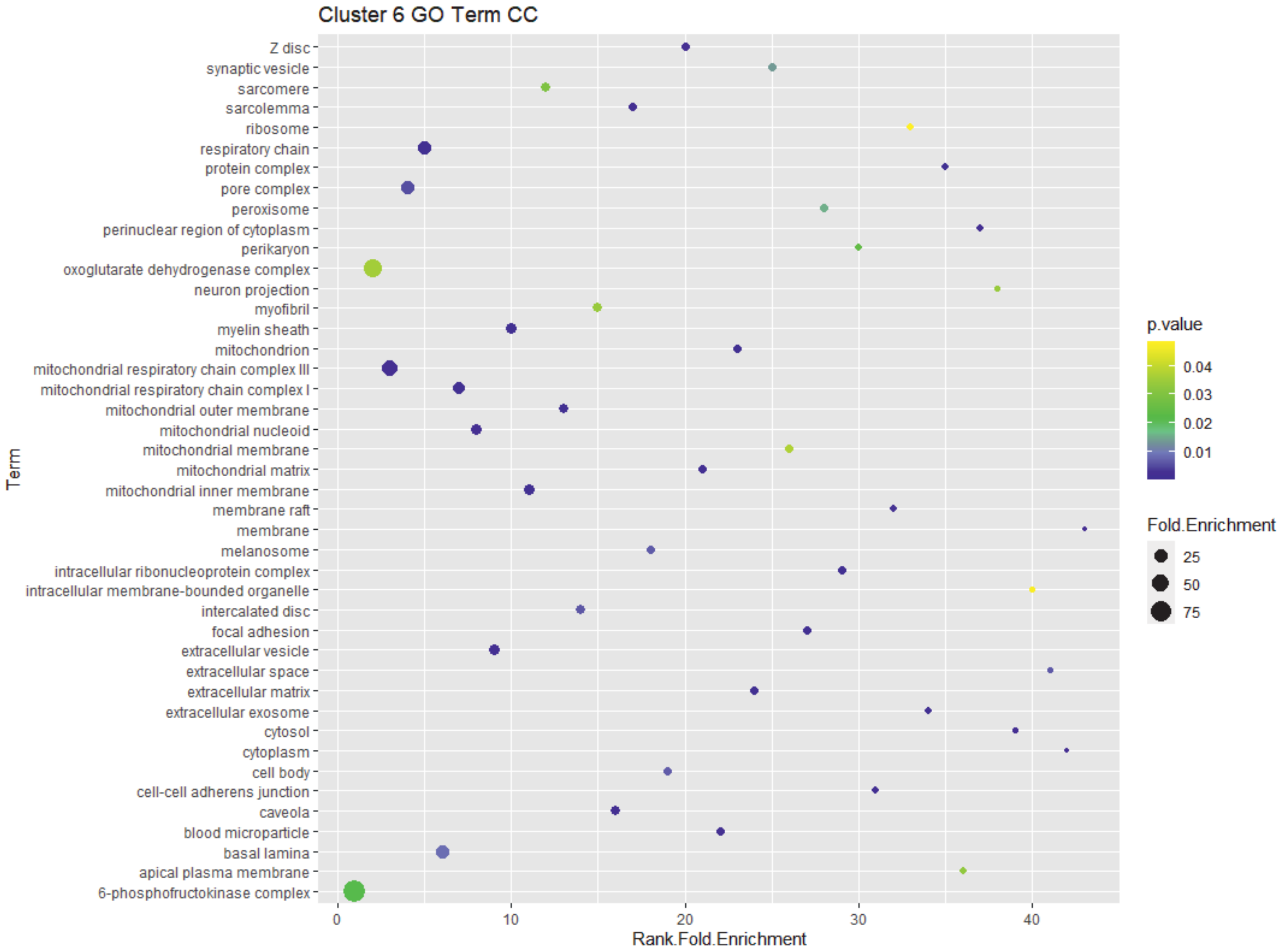
Cluster 6 GO Term CC
Z disc
synaptic vesicle
sarcomere
sarcolemma
ribosome
respiratory chain
protein complex
pore complex
peroxisome
perinuclear region of cytoplasm
perikaryon
oxoglutarate dehydrogenase complex
neuron projection
myofibril
myelin sheath
mitochondrion
mitochondrial respiratory chain complex III
mitochondrial respiratory chain complex I
mitochondrial outer membrane
mitochondrial nucleoid
mitochondrial membrane
mitochondrial matrix
mitochondrial inner membrane
membrane raft
membrane
melanosome
intracellular ribonucleoprotein complex
intracellular membrane-bounded organelle
intercalated disc
focal adhesion
extracellular vesicle
extracellular space
extracellular matrix
extracellular exosome
cytosol
cytoplasm
cell body
cell-cell adherens junction
caveola
blood microparticle
basal lamina
apical plasma membrane
6-phosphofructokinase complex
Term
0
10
20
30
40
Rank.Fold.Enrichment
p.value
0.04
0.03
0.02
0.01
Fold.Enrichment
25
50
75
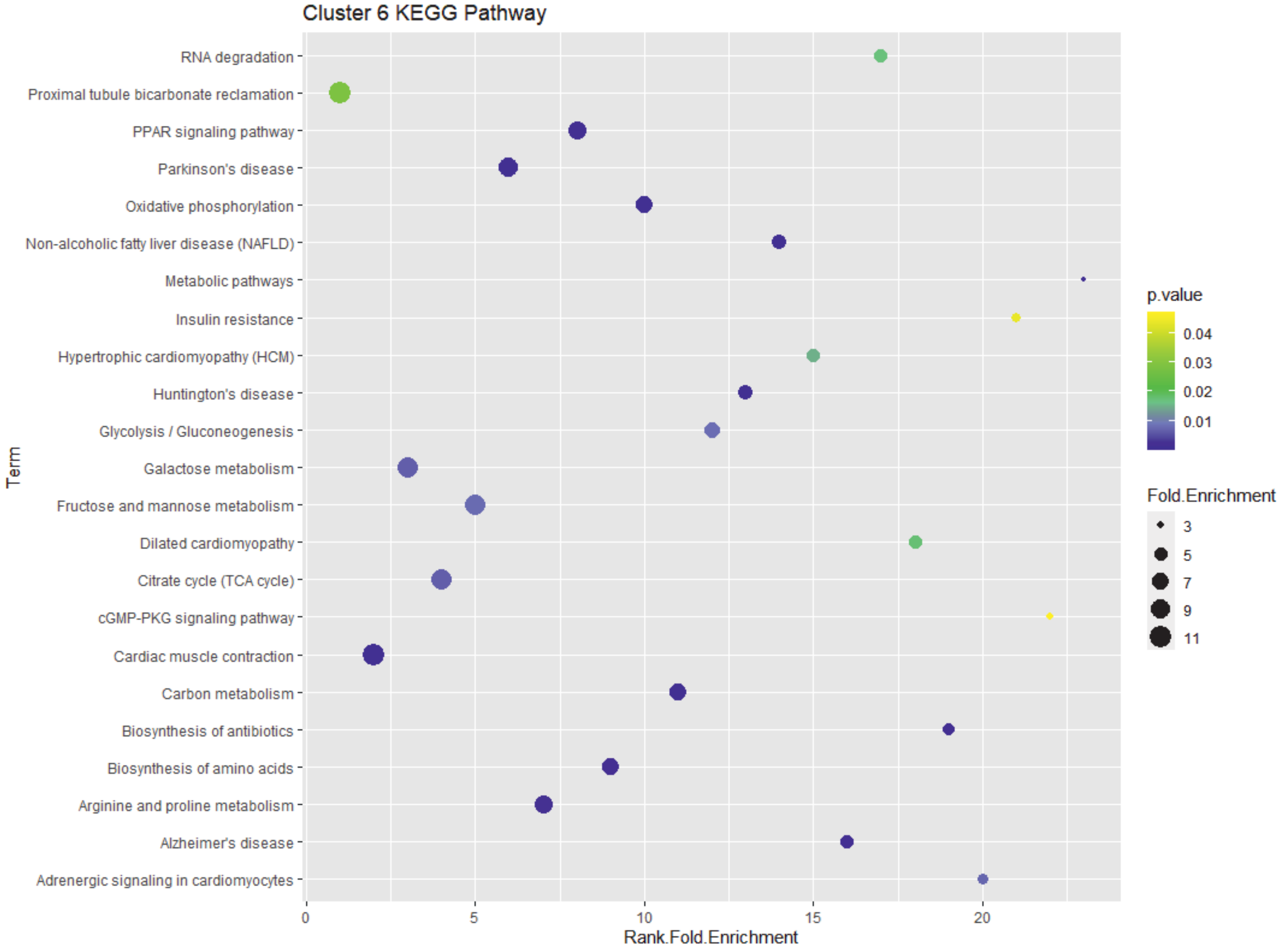
Cluster 6 KEGG Pathway
RNA degradation
Proximal tubule bicarbonate reclamation
PPAR signaling pathway
Parkinson's disease
Oxidative phosphorylation
Non-alcoholic fatty liver disease (NAFLD)
Metabolic pathways
Insulin resistance
Hypertrophic cardiomyopathy (HCM)
Huntington's disease
Glycolysis / Gluconeogenesis
Galactose metabolism
Fructose and mannose metabolism
Dilated cardiomyopathy
Citrate cycle (TCA cycle)
cGMP-PKG signaling pathway
Cardiac muscle contraction
Carbon metabolism
Biosynthesis of antibiotics
Biosynthesis of amino acids
Arginine and proline metabolism
Alzheimer's disease
Adrenergic signaling in cardiomyocytes
Term
0
5
10
15
20
Rank.Fold.Enrichment
p.value
0.04
0.03
0.02
0.01
Fold.Enrichment
3
5
7
9
11
